# Trends in mortality by labour market position around retirement ages in three European countries with different welfare regimes

**DOI:** 10.1007/s00038-012-0359-8

**Published:** 2012-04-29

**Authors:** Seeromanie Harding, Erik Lenguerrand, Giuseppe Costa, Angelo d’Errico, Pekka Martikainen, Lasse Tarkiainen, David Blane, Bola Akinwale, Melanie Bartley

**Affiliations:** 1 Medical Research Council Social and Population Science Unit, University of Glasgow, 4 Lilybank Gardens, Glasgow, G128RZ UK; 2 Department of Clinical and Biological Sciences, Turin University, Turin, Italy; 3 Population Research Unit, Department of Social Research, University of Helsinki, Helsinki, Finland; 4 Imperial College London, London, UK; 5 UK Economic and Social Research Council International Centre for Life Course Studies in Society and Health, University College London, London, UK; 6 Unit of Epidemiology, Regional Health Service, Grugliasco, TO Italy

**Keywords:** Welfare regime, Mortality, Labour market position, International trends

## Abstract

**Objectives:**

In the face of economic downturn and increasing life expectancy, many industrial nations are adopting a policy of postponing the retirement age. However, questions still remain around the consequence of working longer into old age. We examine mortality by work status around retirement ages in countries with different welfare regimes; Finland (social democratic), Turin (Italy; conservative), and England and Wales (liberal).

**Methods:**

Death rates and rate ratios (RRs) (reference rates = ‘in-work’), 1970 s–2000 s, were estimated for those aged 45–64 years using the England and Wales longitudinal study, Turin longitudinal study, and the Finnish linked register study.

**Results:**

Mortality of the not-in-work was consistently higher than the in-work. Death rates for the not-in-work were lowest in Turin and highest in Finland. Rate ratios were smallest in Turin (RR men 1972–76 1.73; 2002–06 1.63; women 1.22; 1.68) and largest in Finland (RR men 1991–95 3.03; 2001–05 3.80; women 3.62; 4.11). Unlike RRs for men, RRs for women increased in every country (greatest in Finland).

**Conclusions:**

These findings signal that overall, employment in later life is associated with lower mortality, regardless of welfare regime.

## Introduction

Increasing the age at which both state-funded and privately funded pensions may be taken is becoming a major response to the present economic crisis in industrial nations. What is often ignored in policy debates around this issue is that many people withdraw from employment or economic activity long before the official retirement ages that are presently in force. Very little research has been done to compare health outcomes of early exit from economic activity in nations with differing policies regarding either retirement or the generosity of income replacement by pensions or other out-of-work benefits. Esping-Andersen’s welfare regime typology distinguishes groups of nations partly in terms of government support during periods without paid employment. In this paper we analyse differences in mortality between those with and without paid employment in later working life over the recent decades (1970s to 2000) in nations that represent the three welfare regimes of Esping Andersen: England and Wales (liberal), Italy (conservative) and Finland (social democratic).

The classification of welfare regimes, using Esping-Andersen’s typology (Bambra 2007; Esping-Andersen 1990, 1996; Ferrera 1996; Leibfried 1993; Rhodes 1996), is based on the extent of de-commodification (the extent to which an individual’s welfare is not reliant upon the market), social stratification (the role of welfare states in maintaining or eliminating social inequality), and the private–public mix (the relative roles of the state, the family and the market) in welfare provision. The liberal model promotes individualism and the primacy of market forces in determining incomes both in and out of employment. The state supports the operation of the market by encouraging private schemes for health, retirement, and sickness and keeping out-of-work benefits to a low level. In the conservative model, the state provides income maintenance closely related to in-work incomes and occupational status, often through organisations that relate to specific occupations. These regimes tend to occur in Catholic nations where more traditional values discourage female labour market participation, in part by the lack of child-care provision. In the social-democratic model, generous income support is provided by the state for those outside of paid employment, with a weaker relationship to previous work history or occupation and not means-tested. Extensive rehabilitative and re-deployment services are available to those unable to work due to ill-health and female employment rates are high.

Different nations classify non-employment in different ways, and the composition of these categories is itself influenced by welfare regime type. In the UK decennial census, the labour market categories of relevance to the ages around retirement are: economically active through either (a) working or (b) job-seeking when unemployed; economically inactive due to either (c) early retirement or (d) permanent sickness or (e) other reasons (mainly full-time home-making).

Our analysis addresses the following questions for those approaching retirement ages: Does the pattern of mortality by labour market position vary between countries with different welfare regimes and have international patterns changed over time? Is the increasing participation of women in the workforce associated with a greater increase in health differences between those in- and not-in-work for women than men? Any such analyses prompt thoughts about the healthy worker effect and questions about health selection into the various labour market categories. We assume that there will be strong health selection into the permanently sick category, possibly significant health selection into the early retired and other inactive categories and some health selection into the unemployed category. Of interest is whether the mortality differentials between the in-work and not-in-work groups differ by welfare regime. These analyses provide indications, of relevance to policy-makers, of how labour market sorting within different welfare regimes may impact on health differentials.

## Methods

### Source of data

Data were extracted from the Office for National Statistics Longitudinal Study (ONS-LS), Turin Longitudinal Study (TLS) and the Finnish linked register study (FS). Information from national censuses was linked to that in death registration records. The FS and the TLS are based on the whole population, and the ONS-LS on a 1 % sample of the English and Wales population. In all the three studies, new members entered via immigration, and existing members left through death or emigration. More details on the design of these studies can be found elsewhere (Cardano et al. 2004; Hattersley and Creeser 1995; Martikainen et al. 2001).

For ONS-LS and TLS, the analysis used socio-demographic information from 1971, 1981, 1991 and 2001 censuses and deaths that occurred in 1972–1976, 1982–1986, 1992–1996 and 2002–2006. For FS, corresponding years for censuses were 1970, 1980, 1990 and 2000, and for deaths were 1971–1975, 1981–1985, 1991–1995, and 2001–2005.

### Sample

State pension age differed across the three countries. In the UK, state pension age is currently 60 and 65 years for women and men, respectively, and 65 years in Finland for both men and women before 2005. For Italy, pensionable age is dependent on age and/or years of contribution, generally 65 for men and 60 for women, but with a minimum age of 55 years. Men aged 50–64 years and women aged 45–59 years were included in the analysis to cover the period before state pension ages in the three countries.

### Explanatory and outcome measures

The labour market information collected at census differs in the three countries, between censuses in the same country, and there are doubts about the comparability of specific categories such as unemployed. Our optimal solution to these problems was to compare those in work around retirement age with an aggregated not-in-work group. In England and Wales, the in-work were those in part-time, full-time employment or self-employment (new categories in 1991 and 2001 censuses); the not-in-work were unemployed (seeking work or waiting to take up a job), government scheme (new in 1991 and 2001) retired, permanently or temporarily sick, independent means, student, looking after home or family, other economically inactive. In Turin, the in-work were those in employment, and the not-in-work were seeking work, retired, student, looking after home (new categories in 1991, 2001), permanently sick (new category 2001) and other. In Finland, the in-work were those classified as employed workforce, and the not-in- work as unemployed workforce, students, doing housework, retired, disabled, other outside workforce, and unknown. Women who were not-in-work were additionally disaggregated into ‘looking after home/family’ or ‘other’. For Finland, this was possible only for 1970 and 1980. Age was grouped in 5-year bands. The outcome measure was all cause mortality. For ease of presentation, the time periods are shown as 1971, 1981, 1991 and 2001.

### Statistical analyses

We examined absolute differences in mortality using death rates and relative differences in mortality using death rate ratios (RRs). Death rates were directly age-standardised using the European standard population from the year 2000. Rate ratios used death rates of those “in-work” as the reference rate. Gender-specific Poisson regression models were used to compare the differences in death rates and in RRs by labour market position within and across countries. Statistically significant effects and interactions (*P* < 0.05) were identified with log-likelihood ratio test. Only significant results are discussed.

Differences between countries were first examined for each time period separately. Country-specific death rates were compared using countries as dummy variables, and differences in RRs using interaction terms of country × labour market position. Within countries, trends in death rates were examined by introducing the census year as the time indicator (continuous variable 0 for first census, 10 for next census etc.) in models, and trends in RRs using labour market position × time interactions. Non-linear time trends were modelled using polynomial functions of time when required. Trends in death rates and RRs across countries were examined using interaction terms of country × labour market position × time.

We also examined age-specific trends within countries using age × labour market position × time interaction terms in country-specific analyses, and the effect of age between time points across countries using country × labour market position × time interaction terms in age-group specific analysis.

## Results

**Fig. 1:**
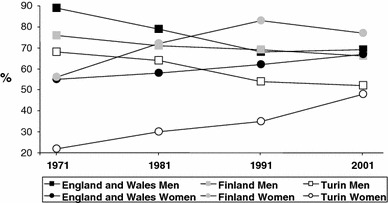
Men and women in work (derived as a percentage of total person-years in a specific country and census) by census year, in England and Wales, Finland, and Turin; 1970/71–2000/01 (1970, 1980, 1990 and 2000 censuses for Finland; 1971, 1981, 1991 and 2001 censuses for England and Wales and Turin; 1971–1975, 1981–1985, 1991–1995, and 2001–2005 mortality data for Finland; 1972–1976, 1982–1986, 1992–1996 and 2002–2006 mortality data for England and Wales and Turin). Sources: ONS-LS, Turin Longitudinal Study, Finnish linked register study

The percentage of men classified as not-in-work increased in all countries, and remained highest in Turin (Table 1). England and Wales had the lowest proportion of men not-in-work in every time period with the exception of 1991–1995, but also had the highest increase over time. Among women, the percentage not-in-work decreased in England and Wales and Turin, and decreased in Finland until 1991–1995. The level remained highest in Turin and lowest in Finland. In England and Wales, and Turin the decrease corresponded with a reduction in women “looking after home/family”. Previous studies for Finland for later years have also shown a reduction (Haataja 2006; Starck 1995; Valkonen et al. 1990). Figure 1 shows that the percentage of men in work decreased while that of women increased. From the 1980s, the level of women in work was greatest in Finland. Table 1 shows deaths, person-years, overall mortality and proportion of person-years accounted for by work status for men and women in all the three nations. Overall mortality of men was lowest in Turin where the percentages not-in-work were highest (Table 1). Among women, overall mortality in the first three time periods was lowest in Finland where the percentages not-in-work were lowest. In 2001–2005, however, overall mortality of women in Turin was as low as in Finland, despite having the highest proportion of not-in-work.

**Table 1:** Age-standardised death rates (SDR) (per 10,000) by census year for men and women living in England and Wales, Finland and Turin

	Men			Women		
	England and Wales	Finland	Turin	England and Wales	Finland	Turin
1971^a^						
SDR (95 %CI)^b^	212 (206, 219)**	199	187**	73 (69, 76)**	46	56
Deaths	4,606	32,233	6,640	1,796	9,544	2,552
Person-years	209,641	1,571,756	341,307	240,842	2,066,082	454,606
Not-in-work^c^	11***	24	32***	45***	44	78***
Looking after home/family^c^				38***	34	68***
Other^c^				7***	10	10***
1981^a^						
SDR (95 %CI)	175 (169, 181)**	159	153*	60 (57, 63)**	35	43**
Deaths	3,661	28,089	6,011	1,411	7,458	2,405
Person-years	204,849	1,759,283	413,191	225,934	2,079,875	555,983
Not-in-work	21***	29	36***	42***	28	70***
Looking after home/family				35***	14	58***
Other				6***	14	12***
1991^a^						
SDR (95 %CI)	131 (126, 136)**	120	112**	45 (42, 48)**	32	35*
Deaths	2,756	23,639	5,154	1,010	7,226	1,789
Person-years	203,529	1,933,501	448,659	222,758	2,340,806	503,711
Not-in-work	32***	31	46***	38***	17	65***
Looking after home/family				25		48
Other				14		17
2001^a^						
SDR (95 %CI)	94 (90, 98)	96	79**	39 (36, 41)**	29	29
Deaths	2,176	24,836	3,304	1,016	8,670	1,282
Person-years	231,816	2,612,562	395,520	261,938	2,911,851	427,670
Not-in-work	31***	34	48***	33***	23	52***
Looking after home/family				18		33
Other				15		19

Sources: ONS-LS, Turin Longitudinal Study, Finnish linked register study

* *P* < 0.001; ** *P* < 0.0001, rate statistically different from the rate in Finland; *** *P* < 0.0001, proportion statistically different from the proportion found in Finland

^a^1970, 1980, 1990 and 2000 censuses for Finland; 1971, 1981, 1991 and 2001 censuses for England and Wales and Turin; 1971–1975, 1981–1985, 1991–1995 and 2001–2005 mortality data for Finland; 1972–1976, 1982–1986, 1992–1996 and 2002–2006 mortality data for England and Wales and Turin

^b^Age-standardised death rate (SDR) and 95 % confidence interval (CI) (WHO-Europe reference) (×10,000). For Finland and Turin, the rates are calculated on the entire population

^c^Derived as a percentage of total person-years, the sum of in-work and not-in work = 100 %

### Absolute and relative mortality differences within and between countries

**Fig. 2:**
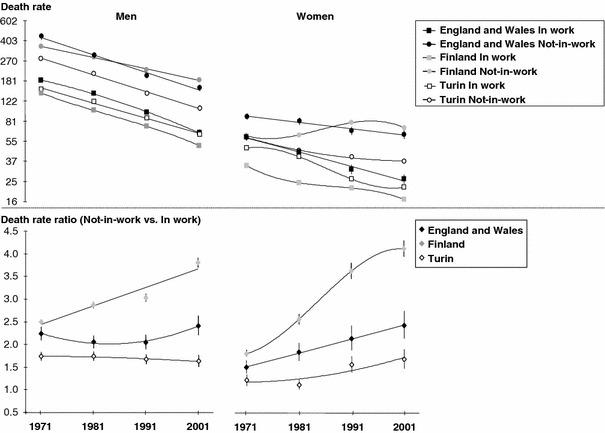
Age-standardised death rates (1970, 1980, 1990 and 2000 censuses for Finland; 1971, 1981, 1991 and 2001 censuses for England and Wales and Turin; 1971–1975, 1981–1985, 1991–1995, and 2001–2005 mortality data for Finland; 1972–1976, 1982–1986, 1992–1996, and 2002–2006 mortality data for England and Wales and Turin) and rate ratios by gender and labour market position in England and Wales, Finland, and Turin between 1971/2 and 2001/2 (Age-standardised death rate (×10,000) (WHO-Europe reference)-Logarithmic scale). Sources: ONS-LS, Turin Longitudinal Study, Finnish linked register study

Figure 2 shows age-standardised death rates and rate ratios (reference = death rates for those in-work) by time periods. In all three countries for both men and women, death rates remained higher for those not-in-work than those in-work. Death rates for the not-in-work were lowest in Turin and the rates for the in-work were lowest in Finland.

Death rates generally fell, with the striking exception of Finnish not-in-work women. In England and Wales, and Italy, death rates for men declined at a similar pace by work status. For women in all three countries and for men in Finland, slower declines in death rates for the not-in-work than for the in-work led to an increase in absolute mortality differences over time. Absolute mortality differences between the in- and not-in-work remained smallest in Turin and largest in Finland in the last two time periods.

Among both men and women, RRs were smallest in Turin and largest in Finland at every time-period. Among men, there was a linear increase in RRs in Finland, an overall small increase in England and Wales, and no increase in Turin. Among women, RRs increased in all three countries, and the increase was greatest in Finland. Within every country, the increase in relative mortality differences between those in work and not in work was greater among women than men, and in 2000/2001–2005 relative mortality was greater in women than in men.

### Age trends in mortality differences

**Fig. 3:**
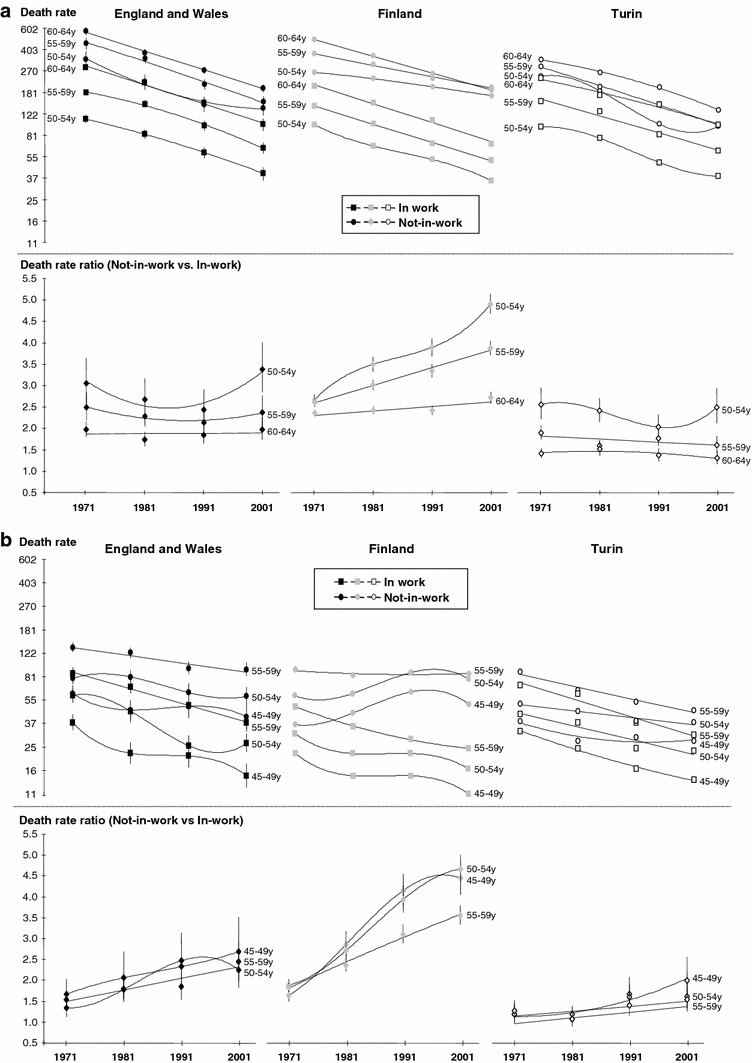
**a** Men-age-standardised death rates (1970, 1980, 1990 and 2000 censuses for Finland; 1971, 1981, 1991 and 2001 censuses for England and Wales and Turin; 1971–1975, 1981–1985, 1991–1995 and 2001–2005 mortality data for Finland; 1972–1976, 1982–1986, 1992–1996 and 2002–2006 mortality data for England and Wales and Turin) and rate ratios by gender, age and labour market position in England and Wales, Finland and Turin between 1971/2 and 2001/2 (Age-standardised death rate (×10,000) (WHO-Europe reference)-Logarithmic scale). Sources: ONS-LS, Turin Longitudinal Study, Finnish linked register study. **b** Women-age-standardised death rates (1970, 1980, 1990 and 2000 censuses for Finland; 1971, 1981, 1991 and 2001 censuses for England and Wales and Turin; 1971–1975, 1981–1985, 1991–1995, and 2001–2005 mortality data for Finland; 1972–1976, 1982–1986, 1992–1996, and 2002–2006 mortality data for England and Wales and Turin) and rate ratios by gender, age and labour market position in England and Wales, Finland and Turin between 1971/2 and 2001/2 (Age-standardised death rate (×10,000) (WHO-Europe reference) − Logarithmic scale). Sources: ONS-LS, Turin Longitudinal Study, Finnish linked register study

Figure 3a (men), b (women) shows age-standardised death rates and RRs (reference = death rates for those in-work) by time periods and age. There was little deviation by age in the general pattern of death rates being the lowest for the not-in-work in Turin and the in-work in Finland. In Finland, the death rate for the not-in-work women aged 45–54 increased between 1970s and 1990s. This was the major contribution to the overall rise in death rates for women not-in-work in Finland (shown in Fig. 2), and to the sharp increase in relative mortality differences between the in- and not-in work groups.

The steeper increases in RRs for men and women in Finland, and generally smaller RRs at every time period in Turin (shown in Fig. 2) were also consistent across age. In Finland, the increase in relative differences was greater in the younger age groups among both men and women.

### Mortality variations within women classified as not employed

Table 2 shows age-standardised death rates and RRs for women classified as ‘looking after home/family’ or ‘other’ not-in-work by time periods.

**Table 2:** Age-standardised death rates and rate ratios by census year not-in-work women living in England and Wales, Finland and Turin; 1970/71–2000/01

Period	Country	Looking after home/family			Other		
		% PY^b^	SDR (95 %CI)^c^	RR (95 %CI)^d^	% PY^b^	SDR (95 %CI)^c^	RR (95%CI)^d^
1971–1975^a^	England and Wales	85	76 (70, 81)	1.28 (1.16, 1.41)	15	164 (143, 184)	2.72 (2.37, 3.11)
	Finland	78	42	1.30 (1.24, 1.36)	22	141	4.04 (3.84, 4.24)
	Turin	87	54	1.15 (1.03, 1.28)	13	101	1.75 (1.52, 2.01)
1981–1985^a^	England and Wales	85	69 (63, 74)	1.56 (1.39, 1.75)	15	148 (129, 168)	3.30 (2.83, 3.84)
	Finland	50	28	1.18 (1.09, 1.27)	50	100	3.90 (3.71, 4.10)
	Turin	83	41	1.05 (0.95, 1.15)	17	70	1.53 (1.34, 1.73)
1991–1995^a^	England and Wales	64	46 (41, 52)	1.50 (1.28, 1.75)	36	105 (93, 116)	3.25 (2.81, 3.77)
	Finland				100^e^	79^e^	3.62 (3.46, 3.80)^e^
	Turin	73	36	1.45 (1.29, 1.64)	27	50 (45, 56)	1.92 (1.67, 2.21)
2001–2005^a^	England and Wales	54	42 (37, 48)	1.66 (1.40, 1.96)	46	86 (77, 96)	3.29 (2.86, 3.79)
	Finland				100^e^	71^e^	4.11 (3.94, 4.29)^e^
	Turin	63	31	1.46 [1.28, 1.67]	37	47	2.07 (1.79, 2.39)

Sources: ONS-LS, Turin Longitudinal Study, Finnish linked register study

^a^1970, 1980, 1990 and 2000 censuses for Finland; 1971, 1981, 1991 and 2001 censuses for England and Wales and Turin; 1971–1975, 1981–1985, 1991–1995 and 2001–2005 mortality data for Finland; 1972–1976, 1982–1986, 1992–1996 and 2002–2006 mortality data for England and Wales and Turin

^b^Person-years of not-in-work women = 100 %

^c^Age-standardised death rate (SDR) and 95 % confidence interval (CI) (WHO-Europe reference) (×10,000). For Finland and Turin, the rates are calculated on the entire population

^d^Death rate ratio and 95 % CI; Reference = In-work

^e^Finland, 1991–2001: Information on looking after home/family not available in Finnish Longitudinal Study. Other study report a very low percentage <5 % (Haataja 2006)

Women classified as ‘looking after home/family’ had lower mortality than those classified as ‘other not-in-work’. The Turinese advantage over the other two countries was evident from both absolute and relative measures for ‘other not-in-work’ women. Compared with Turin or England and Wales fewer women were classified as ‘looking after home/family’ and more were classified as ‘other not-in-work’ in Finland. The death rates for women looking after home/family were much lower in Finland. Other data show that in 2001, less than 3 % of women in Finland were looking after home/family (Haataja 2006), a marked reduction from the earlier decades, suggesting that the not-in-work could be more selected for poor health than women not-in-work in Turin or England and Wales where the large proportions of housewives mask the mortality of those not-in-work.

## Discussion

### Principal findings

Mortality of those not-in-work was higher than those in-work in each time period across welfare regimes. The international differences in relative mortality disadvantage of those not-in-work widened over time. Mortality of those not-in-work was lowest in conservative Turin. By 2001 relative and absolute mortality disadvantage of those not-in-work were greatest in Finland and least in Turin. Increasing female labour force participation appeared to be one reason for the greater increase in relative mortality differences between in work and not-in-work for women than for men in all three countries. The greatest increase occurred in Finland.

Other European studies of welfare regimes and social inequalities in health at working ages have found that Scandinavian welfare regimes do not necessarily show the smallest relative mortality differences (Bambra and Eikemo 2009; Kunst et al. 2005; Mackenbach et al. 1997; Muntaner et al. 2011). The most recently published mortality data refer to 1990s and include younger ages (Mackenbach et al. 2003, 2008). At ages 30–69 years, both relative and absolute differences in total mortality by education or occupational class were greater in Finland than in Turin, and England and Wales generally occupied an intermediate position. In contrast to our findings on labour market categories, these studies on socio-economic inequalities did not report larger mortality differences for women than men (Mackenbach et al. 1999).

### Potential mechanisms

It is difficult to assess the extent to which the temporal trends reported here are influenced by differences in health between those remaining, exiting, or entering the labour market. Selection processes may operate differently across countries, associated with unemployment, sickness and pension arrangements across welfare regimes. In fact one would expect that nations with more generous provision for non-employment might have a higher proportion of non-employed among those in less good health. However, the more generous social democratic welfare regimes also tend to have ‘active labour market policies’ which emphasise rehabilitation and inclusion of those with some degree of disability. They also tend to include far more women with children in the employed labour force, due to better child-care provision. The imperative to remain in work may also depend on intergenerational transfers of wealth, social support networks and family assistance (Börsch-Supan and Schröder 2011).

The composition of the not-in-work group clearly differed across countries. For example in 2001, although the percentage of women not-in-work was lowest in Finland, a greater proportion of women who were not in work were classified as unemployed (41 %) or retired (34 %) than in the other two countries. In England and Wales 6 % of those not in work were classified as unemployed and 12 % as retired while in Turin the corresponding figures were 8 and 26 %. Although there is the issue of the lack of comparability in classification, it is noteworthy that the mortality RRs of each of these not in work categories versus in work appeared to be greatest in Finland. Furthermore this difference was greatest for women classified as ‘retired’. In 2001, the mortality RRs for retired women were 7.95 (95 % CI 6.63–9.53) for Finland, 1.60 (1.35–1.90) for Turin and 1.42 (1.07–1.91) for England and Wales. The corresponding figures for those classified as ‘unemployed’ were 2.73 (2.27–3.20) for Finland, 2.14 (1.44–3.18) for England and Wales and 1.27 (0.93–1.73) for Turin. An increase in labour force participation in women could increase relative mortality differences over time if the proportions not-in-work decreased over time and were increasingly selected for poor health. This may explain some of the increasing differences among Finnish women (Haataja 2006; Starck 1995; Valkonen et al. 1990).

An increase in the proportion not-in-work, as was the case for men, might be expected to be accompanied by a narrowing of mortality differences (Bartley and Plewis 2007; Blane et al. 1999; Martikainen and Valkonen 1996). Relative differences in mortality between men with and without paid employment in Finland, however, increased. At older ages, early retirement due to ill-health is a key factor determining exit from the labour market (Alavinia and Burdorf 2008; Stattin 2005). In Finland, a large proportion of the working-age retired are on disability pensions. The mortality RRs for the retired versus in-work men were higher and increased in Finland (1971 2.70, 95 % CI 2.47–2.95; 2001 4.38, 3.48–5.53) compared with Turin (1971 1.73, 1.63–1.88; 2001 1.40, 1.28–1.52) or England and Wales (1971 1.94, 1.61–2.34; 2001 1.46, 1.24–1.73). The greater increase in mortality differences among Finnish men, compared to Turin, cannot be explained by differences in the changes of the proportion of the not-in-work, which had a similar increase from 1971 to 2001, nor by an increase in the proportion on disability pensions in Finland, which decreased between 1981 to 2001. It is also difficult to reconcile the mortality advantage for the in-work in Finland with policies which promote employment of disabled people. From the late 1990s, compared with Italy or the UK, the percentage of people receiving disability benefits and percentage of disabled people in employment were higher in Finland (OECD 2010).

We used welfare regime an indicator of political traditions and welfare state characteristics that might shape population health. The assumption was that if population health differs across welfare regimes, this may reflect both the political forces and public policies that affect health. Although our approach overcomes the weaknesses of previous studies (large and national, longitudinal, mortality not self reported health etc.), the data available are insufficient to explore socio-cultural or historical trajectories in political or economic traditions.

It is plausible that Western European welfare regimes have changed due to the effect of globalised financial markets on domestic economies (Rhodes 1996). The transfer in manufacturing to Eastern European countries and in information technology jobs to Asian countries may have had a disproportionate impact on the livelihood of manual workers. In the early 1990s, Finland experienced substantial declines in GDP and dramatic increases in unemployment and long-term joblessness. The policy response included a cut in replacement ratios (the value of benefits relative to the average wage). In the UK, there has been a shift in welfare systems away from an emphasis on non-means-tested universal benefits to a system far more directed to lower income groups. Although the UK post-1980 conforms to many characteristics of the liberal regime, it retains universal coverage of health services via a National Health Service which may be one reason for the low mortality of those who are reliant on low levels of benefit income when non-employed. It is also possible that the family-centred culture of conservative Turin may have helped to mitigate the effects of not being in-work, but increasing labour force participation by women raises the question of whether this not-in-work mortality advantage may erode in future.

### Strengths and weaknesses

The use of longitudinal studies and of mortality as an outcome avoids the biases inherent from cross-sectional data or self assessed reports of health. The reliability of the estimates is also underscored by large sample sizes, minimal item non-response and low loss to follow-up. However, any single country may not represent a welfare regime well and the findings for Turin cannot be generalised to all Italy.

Longitudinal analyses of changes in labour market position at the individual level could have shed some light on the extent to which exit from the labour market might have been due to ill-health. In Italy, 52 % of women looking after home/family at 2001 census, and present at all four censuses, had been previously employed. Cause-specific mortality could have provided clues about specific behavioural risk factors. Social inequalities in cardiovascular mortality are smaller in Italy than in Finland and in the UK (Kunst et al. 1998a, b, c). In Finland, in the early 1990s the excess mortality among 25–59-year-old unemployed stemmed from alcohol-related diseases among both men and women and among men also from respiratory diseases, accidents and violence (Martikainen and Valkonen 1995). Among men these causes were also mostly responsible for the disparity already in the early 1980s (Martikainen 1990). Social inequalities in smoking and smoking-related mortality also differ between countries and periods; smaller inequalities are observed for Italian men and a reverse gradient for Italian women (Huisman et al. 2005; Mackenbach et al. 2008). The latter raises the question of the extent to which smaller/reverse social gradients in risk factors contributed to the smaller mortality differences between in work and not-in-work groups in Turin.

Early withdrawals from the labour market due to uptake of disability pensions are more likely to be from manual social classes. In the manual classes entry to the labour market is at a younger age and, in countries where pension age depends on length of service, this is achieved earlier than their non-manual peers. We could not reliably examine the effect of differences in socio-economic composition on the mortality differences between in-work and not-in-work between countries with these data. In England and Wales, before 2001, the question on education identified those with or without higher education (the overwhelming majority classified as without), and income has never been collected in any of the censuses. A possible comparable measure was occupational class but large proportions of the not-in-work in England and Wales or Turin were not allocated to social class in the earlier decades. In England and Wales, it was not until the 1991 census that current or previous occupation was reported regardless of current employment or health status. In Turin, class was assigned only to those in work and if not in work then information from previous censuses was used. Among men not-in-work in England and Wales, 40 % were not classified in 1971, 20 % in 1981, 28 % in 1991 and 11 % in 2001. The comparable proportions in Turin were 100 % in 1971, 26 % in 1981, 15 % in 1991 and 17 % in 2001. These proportions were larger for women even after assigning husband’s class or class from information in previous census. However, bearing these caveats in mind, adjusting the RRs for not-in-work versus in-work for social class did not affect the between-country differences. For e.g., in 2001–2005, among men the RRs adjusted for class were as follows: Finland 3.70 (95 % CI 3.52–3.88), England and Wales 2.23 (2.04–2.44), Turin 1.63 (1.50–1.77). In 2001–2005, among women the adjusted RR for Finland for all not-in- work was 4.10 (3.80–4.43), and the RRs for England and Wales looking after home/family 1.54 (1.30–1.82), other not-in-work 3.01(2.61–3.48), and for Turin looking after home/family 1.41 (1.23–1.62) and other not-in-work 2.06 (1.79–2.38).

### Conclusion

Long-term trends in work status and mortality around retirement age in different welfare regimes have not been reported before. Mortality of the not-in-work was highest in Finland and lowest in Turin, and these differentials increased over time. Women’s increasing employment may account for a trend of rising relative mortality disadvantage of those not-in-work. However, it remains difficult to disentangle whether these mortality differentials reflect varying and changing selection effects or possibly differentials in the working of the welfare regimes with regards to replacement ratios. Welfare regime theory relates mostly to working life income protection and the allocation of welfare within societies; perhaps the altered ageing and gender contexts of health at the end of working life require equal emphasis in welfare theory.
